# Optimising reliability of mouse performance in behavioural testing: the major role of non-aversive handling

**DOI:** 10.1038/srep44999

**Published:** 2017-03-21

**Authors:** Kelly Gouveia, Jane L. Hurst

**Affiliations:** 1 Mammalian Behaviour and Evolution Group, Institute of Integrative Biology, University of Liverpool, Leahurst Campus, Neston CH64 7TE, UK

## Abstract

Handling laboratory animals during test procedures is an important source of stress that may impair reliability of test responses. Picking up mice by the tail is aversive, stimulating stress and anxiety. Responses among anxious animals can be confounded further by neophobia towards novel test environments and avoidance of test stimuli in open areas. However, handling stress can be reduced substantially by using a handling tunnel, or cupping mice without restraint on the open hand. Here we establish whether non-aversive handling, brief prior familiarisation with the test arena and alternative stimulus placement could significantly improve performance of mice in behavioural tests. We use a simple habituation-dishabituation paradigm in which animals must discriminate between two urine stimuli in successive trials, a task that mice can easily perform. Tail handled mice showed little willingness to explore and investigate test stimuli, leading to poor test performance that was only slightly improved by prior familiarisation. By contrast, those handled by tunnel explored readily and showed robust responses to test stimuli regardless of prior familiarisation or stimulus location, though responses were more variable for cup handling. Our study shows that non-aversive tunnel handling can substantially improve mouse performance in behavioural tests compared to traditional tail handling.

Environmental stressors within the laboratory are a well-recognised source of unexplained background variation that influences the performance of laboratory animals in behavioural tests. This is because an animal’s performance during testing is determined by its behaviour, which in turn is susceptible to environmental stressors within the laboratory^1^. This can create considerable problems for replicating responses across batches of animals, experiments and laboratories, and for direct comparison of responses between studies^2^,^3^. Perhaps even more important than the effects on increased variability, unnecessary stress or anxiety during testing is likely to shift the animal’s attention away from a particular test or stimulus and impair its ability to learn and/or solve specific tasks. This may thereby impair the reliability of test subjects and potentially provide misleading data that might appear consistent, but reflects an inappropriate interaction with the test rather than a performance measure that the test was designed to assess.

The handling of laboratory animals during testing is a widely recognised source of stress that needs to be controlled, particularly as stress induced by handling can suppress exploratory behaviour and result in impaired test performance^4^,^5^. In mouse behavioural phenotyping studies, for example, handling stress has been identified as one of the most likely causes of failure in replicating phenotypes within and between experiments^3^,^6^. However, the general solution proposed to deal with this issue has been to standardize handling procedures or, where possible, minimise handling involved in testing^7^,^8^, rather than consider how to reduce or remove the impact of handling on stress and anxiety to eliminate any interference with the test. We now know that the method used to handle laboratory mice has a substantial impact on their willingness to interact with handlers and the anxiety shown in standardised tests^9^,^10^,^11^. Mice picked up by the base of the tail are very unwilling to interact voluntarily with handlers and show high anxiety in elevated plus maze tests, even when highly familiar with this traditional handling method. By contrast, mice tame quickly when picked up using a handling tunnel, or are accustomed to being cupped on the open hand without direct physical restraint. They show low levels of anxiety and will readily approach the handler. Thus, choice of handling method is likely to have a significant influence on the welfare of laboratory mice^12^, but the consequences of handling method for the responses shown in scientific testing remain to be established. As tail handling is aversive to mice and stimulates anxiety compared to the alternative non-aversive handling methods, we hypothesized that use of non-aversive handling could significantly improve the performance of animals in behavioural tests by reducing or removing unnecessary anxiety that may interfere with attention to test stimuli and/or learning.

Here, we test this hypothesis using a simple habituation-dishabituation paradigm that is commonly used to test discrimination between two stimuli e.g. refs 13, 14, 15, 16. In this test, animals are given a series of trials in which an initially novel stimulus is presented repeatedly in successive trials to induce reduced investigation as the stimulus becomes familiar (habituation response). A different stimulus is then presented to assess whether animals discriminate a difference between the new and previous stimulus, evident from increased investigation of the new stimulus (the dishabituation response). Using this test allowed us to examine the initial response of mice to a novel attractive stimulus (urine from the opposite sex) that should stimulate approach and investigation^17^, after animals had experienced handling and delivery to a novel test arena by different methods. It also allowed us to examine the longer-term effects on habituation and dishabituation responses when repeatedly delivered to the arena by aversive or non-aversive handling. Test responses depend on the subject’s attention to the stimuli combined with simple learning and discrimination abilities, which are susceptible to interference from exploratory behaviour in a novel environment and to any reduced motivation to investigate stimuli^15^,^18^,^19^. Negative influences of handling and novel test environments can sometimes be avoided by conducting tests within individual home cages e.g. refs 20, 21, 22. However, our test design maximized the potential for interference by testing animals in a clean arena with repeated handling to contrast the impact of aversive and non-aversive handling methods. Other factors of test design, such as prior familiarity with the test arena and the location of stimulus presentation, may also influence the interaction of animals with test stimuli. Therefore we conducted two separate experiments to look at the combined influence of handling method and stimulus location (experiment 1), or handling method and prior familiarity with the test arena (experiment 2). We show that the use of tail handling creates a substantial interference with test responses, as animals failed to show free exploration of the test arena or pay sufficient attention to urine test stimuli. This led to very poor performance measures in a test that should have been easy to perform. By contrast, mice handled by non-aversive methods showed ready exploration and strong test performance regardless of the stimulus location or prior familiarisation with the test arena.

## Experiment 1: Effects of Handling Method and Stimulus Location

When placed into an unfamiliar open field arena, anxious mice show reduced movement around the arena, and are reluctant to enter or spend time in the open, unprotected central area^23^,^24^,^25^. Indeed, open field tests are often used alongside other tests to assess anxiety-related behaviour^1^,^26^. However, when the purpose of a test is to assess response to a specific stimulus presented in a test arena rather than assessment of anxiety, an anxiety-related reduction in exploratory behaviour is likely to impair interaction with the stimulus. Further, as anxious animals are less likely to visit open areas, the location of the stimulus may also be critical. Presenting stimuli closer to an arena sidewall rather than in the centre might be much more effective in facilitating a subject’s interaction with the stimulus on which the test depends. In this experiment, we manipulated both the handling method that subjects experienced and the location of an attractive stimulus (in the centre or periphery), to establish whether either or both of these factors improved the performance of naïve mice in an olfactory habituation-dishabituation test. We compared mice handled by the traditional tail method with those handled by two non-aversive methods: use of a handling tunnel familiar from the subject’s home cage or cupping on the open gloved hand. In previous studies, we had shown that accustoming mice to one of these alternative handling methods through 60 s daily handling over several days led to substantially lower anxiety-related behaviour compared to those handled by the tail^9^,^10^. Extensive handling of subject animals prior to the commencement of behavioural testing is often used to reduce stress associated with handling e.g. refs 27 and 28, but this is time consuming, impractical for large numbers of animals and may not be effective if aversive methods are used. In this experiment, subject mice experienced only brief handling, using their assigned method, to transfer them between cages during bimonthly routine cage cleaning (from 5 to 14 weeks of age) and during the test procedure itself. We made no attempt to additionally habituate animals through extensive handling prior to testing.

### Experiment 1: Results

#### Effects of handling method and stimulus location

**Figure 1:**
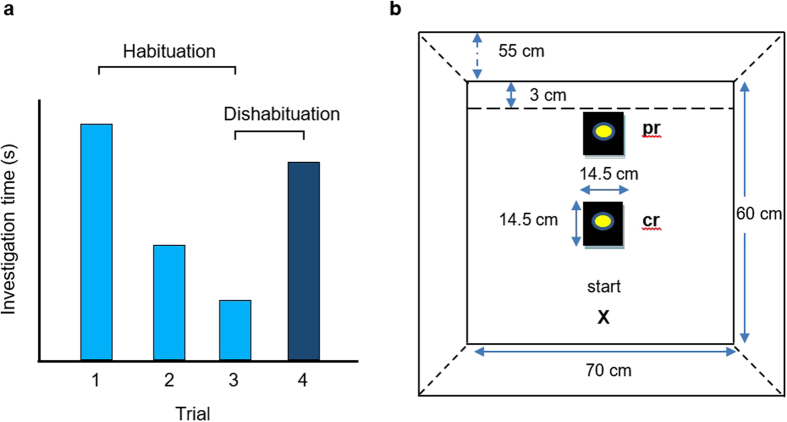
The habituation-dishabituation task. (**a**) Principle underlying the habituation-dishabituation task. Repeated presentation of a novel stimulus for three successive trials (light blue bars) induces a reduction in investigation as the animal recognises an increasingly familiar stimulus (habituation, measured as less investigation in trial 3 compared to trial 1). When an unfamiliar stimulus is presented on the fourth trial (dark blue bar), investigation increases if the animal recognises that the stimulus is novel (dishabituation, measured as more investigation in trial 4 than trial 3). (**b**) Test arena, showing the location of a urine stimulus either at the centre (cr) or periphery (pr) of the arena. Mice were introduced at the lower edge of the arena (start location marked with X). Animals handled with a tunnel were tipped out backwards^9^ at floor level. Urine stimuli used in trials 1–4 came from unrelated male mice.

**Figure 2:**
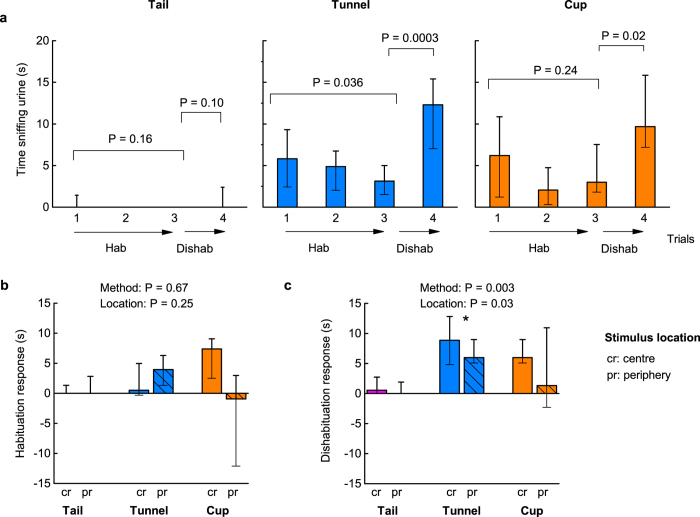
Handling method and stimulus location influences test performance in a habituation-dishabituation task. In experiment 1, time sniffing a urine stimulus was recorded in successive trials (1–4), with urine derived from one male in trials 1–3 and from a different male in trial 4. Mice were handled by tail (pink), tunnel familiar from the home cage (blue) or cupped on the hand (orange). Data are medians ± interquartile range. (**a**) Wilcoxon signed ranks tests assessed habituation (reduced stimulus investigation of the same scent in trial 3 compared to trial 1), and dishabituation (greater investigation of novel scent in trial 4 compared to familiar scent in trial 3). Tests with the stimulus in different locations are pooled. Comparison of (**b**) habituation responses (trial 1-trial 3 investigation) and (**c**) dishabituation responses (trial 4-trial 3 investigation) according to handling method and stimulus location (arena centre: cr, solid bars; periphery: pr, hatched bars). *P* values from likelihood ratio tests comparing mixed effects models (Table 1). N sizes: Tail cr (8), pr (8); Tunnel cr (8), pr (7); Cup cr (6), pr (7).

In our habituation-dishabituation task, each animal received three 5 min trial presentations of the same urine stimulus to induce reduced sniffing of the stimulus, measured as a reduced response in trial 3 compared to trial 1 (the habituation response). A different urine stimulus was then presented in the fourth trial to induce dishabituation (i.e. greater response in trial 4 compared to trial 3, see Fig. 1a). Overall, the habituation response between the first and third trials was relatively weak but statistically significant when pooled across all treatment groups (Wilcoxon matched-pairs test, z = 2.11, P = 0.02; Fig. 2a). Linear mixed effects models (taking home cage into account as a random effect, Table 1a) showed that neither handling method (tail, tunnel or cup: P = 0.67) nor stimulus location (centre or periphery of the arena: P = 0.25) significantly influenced this weak response (Fig. 2b). By contrast, dishabituation when presented with a new urine stimulus on the fourth trial was strong overall (Wilcoxon matched-pairs test, z = 4.16, P < 0.0001; Fig. 2a), but this depended both on the handling method (P = 0.003) and, to a lesser extent, on stimulus location (P = 0.03, Table 1a; Fig. 2c). Dishabituation responses were stronger when the stimulus was located in the centre of the arena rather than at the periphery (Fig. 2c). Dishabituation was also considerably more robust among mice handled with a tunnel compared to those handled by tail (Fig. 2c), with all those handled by tunnel sniffing the stimulus longer in trial 4 than in trial 3. Mice cupped on the hand also showed stronger dishabituation than tail handled mice, though the response tended to be more variable than for tunnel handled mice (Fig. 2c). Notably, within each handling method, only mice handled by tunnel achieved a significant test performance on both the habituation and dishabituation stages of the test (n = 15 mice). Tail handled animals did not reach significance for either stage (n = 16 mice), with most failing to sniff the stimulus even once in each of the four trials (Fig. 2a).

#### Relationship between exploratory behaviour and test performance

**Figure 3:**
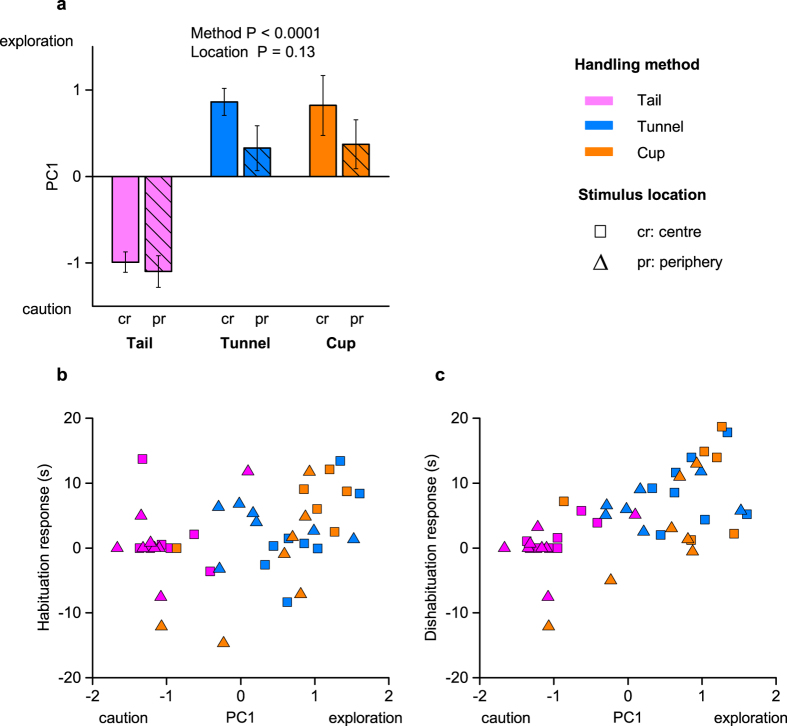
Effects of handling method and stimulus location on exploratory behaviour in experiment 1. PCA extracted a single component (PC1) that accounted for 75% of variance in behaviour averaged over four successive trials for each subject. This contrasted high positive weights for active exploration (movement around the arena: 0.94, visits to the stimulus: 0.92, time sniffing stimulus: 0.83) with negative weighting for cautious behaviour (frequency of stretched attend postures: −0.77). (**a**) Effects of handling method and stimulus location on PC1 scores (means ± sem), *P* values from likelihood ratio tests comparing mixed effects models (Table 1). Correlation between exploratory behaviour and (**b**) habituation (stimulus investigation in trial 1-trial 3), or (**c**) dishabituation (stimulus investigation in trial 4-trial 3). The stimulus was placed in the centre (squares) or periphery (triangles) of the arena and animals were handled by tail (pink), tunnel familiar from the home cage (blue) or cupped on the open hand (orange). N sizes as in Fig. 2.

To assess general exploratory behaviour during testing, we averaged behaviour over all four trial presentations. Principal components analysis (PCA) extracted a single component (PC1) that accounted for 75% of variance in mouse behaviour. PC1 contrasted behaviours reflecting active exploration that were positively correlated (movement around the arena, number of visits to the stimulus tile and time sniffing the stimulus) with the frequency of static stretched attend postures, a cautious risk assessment behaviour associated with anxiety. Handling method had a highly significant influence on general exploratory behaviour during testing: mice handled using a tunnel or cupped on the open hand exhibited much more active exploration and less caution than those that were picked up by the tail (P < 0.0001, Table 1a; Fig. 3a). The location of the stimulus was not a significant predictor of this exploratory behaviour (P = 0.13, Fig. 3a).

As handling method had such a major influence on general exploratory behaviour, we investigated whether adding the level of exploratory behaviour to our model, together with handling method and stimulus location, significantly predicted habituation and dishabituation responses (Table 1b). General willingness to explore the arena was a strong predictor of performance during testing: animals with higher levels of active exploration (PC1 scores) showed stronger habituation responses (P = 0.008; Fig. 3b) and much more pronounced dishabituation responses (P = 0.005; Fig. 3c). Further, when exploratory behaviour was included in the model, there were no additional effects of handling method on dishabituation (P = 0.46) or habituation (P = 0.12) responses. The variance in dishabituation responses explained by stimulus location was also slightly reduced (P = 0.08). This suggests that individual level of exploratory behaviour, which was strongly influenced by handling method, was the major predictor of test performance.

### Experiment 1: Discussion

Handling method had a strong influence on the general exploratory behaviour of mice during testing and, in turn, this was a major predictor of dishabituation test performance. Those picked up in a tunnel or cupped on the hand showed much more active exploration during testing than those picked up by the traditional tail method. Consequently, they showed much greater awareness or interest when a novel stimulus was introduced. Indeed, tail handled mice showed very low exploration and many of these animals failed to investigate the stimulus even once. As handling anxiety reduced exploration generally, placing the stimulus in the periphery did not improve response to the test stimuli. Indeed, dishabituation responses were a little stronger when the stimulus was placed centrally, due to a greater contrast in investigation of the familiar and novel stimulus among tunnel and cup handled mice that explored the arena more freely.

General exploratory behaviour was also a significant predictor of the habituation response. However, although mice handled by tunnel or cupping showed much more active exploration, only those with very high exploration scores showed consistent habituation responses, resulting in no significant effect of handling method on the initial habituation phase of the test. Examination of the data indicated that this may be because initial investigation of the novel stimulus presented in trial 1 was generally weak, and less than investigation of a new stimulus in trial 4 (across all trials, Wilcoxon matched-pair test, z = 2.74, P = 0.005; Fig. 2a), even though the scent stimulus was novel in both of these trials.

## Experiment 2: Effects of Familiarisation on Mouse Test Performance

We hypothesized that low investigation of a novel stimulus in the first trial, regardless of handling method, could be due to the unfamiliarity of the test arena. It is well established that familiarising rodents with a test environment can reduce neophobic responses during testing, and may improve performance in cognitive tasks^29^. However, the impact of familiarisation on behaviour is rarely tested formally in behavioural neuroscience studies, and little information has been published on how much familiarisation is needed to significantly improve test performance. A requirement for extensive familiarisation will add significantly to the time and effort needed to test each individual animal. Further, the effects of familiarisation may depend on the handling method used if both have an influence on anxiety during testing but one has a much stronger effect or counteracts the other. We set up a second experiment to determine whether brief prior familiarisation with the test arena is sufficient to improve performance in a habituation-dishabituation task, and whether handling method influences this. As mice handled using a tunnel showed the most robust test performances in our first experiment while those cupped on the hand were more variable (see also Hurst & West^9^), we compared the responses of mice handled with a tunnel with those handled by the traditional tail method. Mice in half of the cages in each handling group were placed in the test arena for 10 min before testing began to familiarise them with the arena (no urine stimulus was present during this familiarisation period). In this experiment, animals were obtained as adults and familiarised with their assigned handling method prior to testing by picking them up daily for 2 s (equivalent to the duration of handling at cage cleaning) for ten days prior to testing.

### Experiment 2: Results

#### Effects of familiarisation and handling method

**Figure 4:**
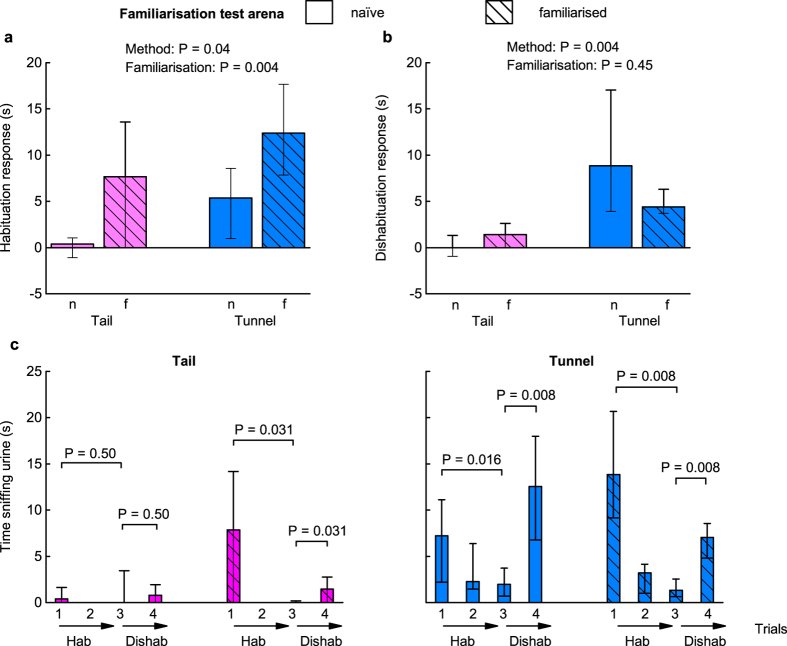
Familiarisation and handling method influence test performance in the habituation-dishabituation task. Comparison of (**a**) habituation (trial 1-trial 3 stimulus investigation) and (**b**) dishabituation (trial 4-trial 3 investigation) according to handling method (pink: tail; blue: tunnel) and prior familiarisation (familiarised: f, cross hatched; naïve: n, solid bars). Data are medians ± interquartile range, *P* values from likelihood ratio tests comparing mixed effects models (Table 2). (**c**) Time sniffing the urine stimulus in successive trials (1–4) for mice handled by tail (pink) or tunnel (blue) when naïve (solid bars) or familiarised (cross hatched bars) with the test arena (medians ± interquartile range). *P* values from Wilcoxon matched pairs exact tests examining habituation (greater stimulus investigation of same scent in trial 1 than trial 3), and dishabituation (greater investigation of novel scent in trial 4 compared to familiar scent in trial 3). N = 8 mice in each method x familiarisation group.

Brief familiarisation with the arena immediately before testing improved habituation responses to the test stimulus across both tunnel and tail handled mice, but those handled by tunnel showed stronger habituation responses than those handled by tail (Fig. 4a). A linear mixed effects model (taking home cage into account as a random effect, Table 2a) confirmed that both familiarisation (P = 0.004) and, to a lesser extent, handling method (P = 0.04) were significant predictors of the habituation response (Fig. 4a). As we hypothesized, this was because investigation of the stimulus in the first trial increased significantly if animals were already familiar with the arena (effect of familiarisation on stimulus investigation in the first trial, χ^2^ (1) = 6.00, P = 0.014) and if they were handled by tunnel rather than tail (χ^2^ (1) = 5.24, P = 0.022; Fig. 4c).

As found in experiment 1, dishabituation responses were strongly dependent on handling method (data log transformed, P = 0.004, Table 2a), with tunnel handled mice showing robust dishabituation compared to tail handled mice (Fig. 4b). Prior familiarisation did not improve dishabituation performance (P = 0.45, Table 2a; Fig. 4b). Thus, familiarisation improved willingness to investigate a stimulus on first introduction to the test arena, improving performance for the habituation stage of the test. However, there was little evidence that this familiarisation had a sustained influence on behaviour over repeated trials. By contrast, handling method influenced both habituation and dishabituation responses. Tunnel handled mice showed robust responses to the test stimuli, even without prior familiarisation; but test responses among tail handled mice were evident only with prior familiarisation and still were much weaker than for tunnel handled mice (Fig. 4c).

#### Relationship between exploratory behaviour and test performance

**Figure 5:**
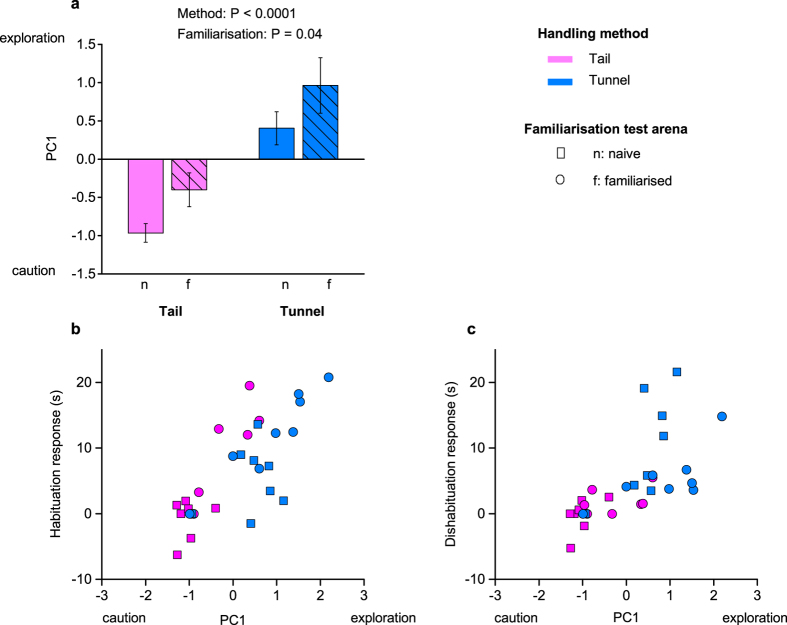
Effects of familiarisation and handling method on exploratory behaviour in experiment 2. PCA extracted a single component (PC1) that accounted for 78% of variance in behaviour averaged over four successive trials. This contrasted high positive weights for active exploration (movement around the arena: 0.95, visits to the stimulus: 0.95, time sniffing stimulus: 0.92) with negative weighting for cautious behaviour (frequency of stretched attend postures: −0.67). (**a**) PC1 scores (means ± s.e.m.) according to handling method and familiarisation (familiarised: f, cross hatched; naïve: n, solid bars). *P* values from likelihood ratio tests comparing mixed effects models (Table 2). Correlation between exploratory behaviour and (**b**) habituation (stimulus investigation in trial 1–3), or (**c**) dishabituation (stimulus investigation in trial 4–3). Mice were either naïve (squares) or familiarised with the test arena (circles) prior to testing, and handled by the tail (pink), or with a tunnel familiar from the home cage (blue). N = 8 in each method x familiarisation group.

As in our first experiment, PCA extracted a single component (PC1) that contrasted active exploration (movement around the arena, stimulus visits and duration of sniffing) over the four trials with cautious risk assessment behaviour, accounting for 78% of the variance in behaviour. Handling method had a major influence on this willingness to explore the arena (P < 0.0001, Table 2a), with mice handled using a tunnel exhibiting much higher scores than those handled by the tail (Fig. 5a), in line with experiment 1. Brief familiarisation with the arena prior to testing had a lesser but statistically significant effect on increased exploratory behaviour (P = 0.04, Table 2a).

Including exploratory behaviour (PC1) in our models indicated that this was a significant predictor of test performance that accounted for the influence of handling method (Table 2b). Animals that explored the arena more actively showed stronger habituation responses (effect of including PC1 in the model: P < 0.0001, Table 2b; Fig. 5b). In this model, handling method did not have any additional effect on the habituation response that was not accounted for by inclusion of exploratory behaviour (P = 0.21). However, familiarisation continued to have an additional effect (P = 0.009), indicating that familiarisation improved habituation not simply through the increase it induced in general exploratory behaviour. Increased exploration also led to stronger dishabituation responses (P = 0.02, Table 2b; Fig. 5c), with no additional effects of handling method (P = 0.51) or familiarisation (P = 0.82). When the non-significant effects of method and familiarisation were dropped from the model, the level of active exploration (measured by PC1) was a highly significant predictor of the strength of dishabituation response (χ^2^ (1) = 13.59, P = 0.0002).

### Experiment 2: Discussion

Our second experiment confirmed that briefly familiarising animals with the test arena before starting a test led to increased investigation of the novel urine stimulus on the first trial, which then led to more robust habituation responses. The impact of prior familiarisation with a test arena on improved performance in novel object recognition^30^,^31^,^32^and other tasks^33^ is well established. Many studies recommend the use of extensive prior familiarisation with repeated exposures over many days, but 10 minutes familiarisation immediately prior to testing was effective in improving initial stimulus investigation in the current study. Importantly, though, this mostly influenced tail handled mice, which showed extremely low stimulus investigation across all trials without arena familiarisation (confirming responses in Experiment 1). Most published experiments or phenotyping programmes do not indicate the method used to handle mice for delivery to a test or during background cage maintenance, but tail handling is by far the most common method used e.g. ref. 34. Although tail handled mice behave consistently, it is clear that anxiety induced by tail handling interferes with behavioural test responses, requiring additional procedures to attempt to overcome this. By contrast, mice handled with a tunnel performed well, with or without prior familiarisation to the test arena. They showed consistently greater exploratory behaviour than those handled by tail, which led to reliable investigation of novel urine stimuli (as expected and required by the habituation-dishabituation test). Thus, neophobia when placed in a novel environment appears to be greatly exacerbated by aversive handling. Although prior familiarisation slightly improved investigation on the first trial when handled by tunnel, it did not contribute to an overall improved performance to the test itself.

Notably, even with prior familiarisation, tail handled mice showed very low investigation of a novel stimulus on the fourth ‘dishabituation’ trial. By this stage, animals had experienced repeated handling using an aversive method. It is possible that much more extensive familiarisation of tail handled mice prior to testing could improve this response, but mice do not habituate readily to being picked up by the tail even after many handling sessions^9^,^10^. Thus, increasing exposure to aversive handling before testing, as well as during the test, may increase anxiety and reduce performance, counteracting the benefits of improved familiarity with the test environment itself. By contrast, use of a handling method that is non-aversive to mice can remove the requirement for prior familiarisation with the handling procedure and test environment, as animals that are not anxious will readily explore the novel environment. This could save valuable time during testing, as well as substantially improve the reliability of behavioural responses to test stimuli that are not confounded by handling-induced anxiety.

## General Discussion

Previous research has established that handling mice using a tunnel or cupped on the open hand is non-aversive and reduces anxiety compared to the traditional tail handling method^9^,^10^. Such non-aversive methods can also reduce physiological stress responses to handling that can confound metabolic studies, such as the effects of handling stress on glucose tolerance^11^. The current study examined the extent to which these different handling methods impact on performance in a simple cognitive test that was designed to reveal how handling anxiety might confound behavioural responses to test stimuli. For this purpose, we used a test stimulus (male mouse urine) that is attractive and biologically relevant to female mice, and thus should reliably stimulate investigation in a habituation-dishabituation test. The low exploration and more cautious behaviour shown by tail handled mice in the test compared to those handled by non-aversive methods is fully consistent with the well-established correlation between anxiety in rodents and reduced exploratory behaviour^26^,^35^. It is also fully consistent with our previous findings that tail handled mice show increased stress during handling (frequent urination and defecation), high anxiety in elevated plus maze tests and avoid approaching their handlers. As we predicted, the reduced willingness to explore among anxious tail-handled mice led to very poor investigation of test stimuli (despite the normal attractiveness of male urine) and thus very poor performance in tests. As those handled with non-aversive methods were more willing to explore and showed very good test responses, poor performance was due to the handling method. In this study, animals were handled and tested in the dark phase of the diurnal cycle. However, Hurst & West^9^ demonstrated that non-aversive handling methods had very similar effects on willingness to approach a handler, exploration and anxiety in elevated plus maze tests, and urination and defecation stress measures, whether animals were handled and/or tested in the light or dark phases of the diurnal cycle. Thus, the different handling methods are very likely to have similar effects on exploratory behaviour and test performance in the dark or light, although anxious animals may be even less likely to explore freely when tested in the light.

The general recommendation to reduce handling stress is to habituate animals to frequent contact with the handler prior to testing^36^. To establish the impact of different handling methods on aversion and anxiety in mice previously, we used daily handling for 60 s to ensure that any differences between the methods would be highlighted^9^,^10^. However, in the current study, prior handling experience was restricted to the brief pick-up that animals experience during normal background cage cleaning, when animals are transferred between cages, or equivalently brief (2 s) handling experienced for 10 days. Despite this very brief handling, when willingness to interact voluntarily with the handler was measured before testing began, responses were similar to those found with much more prolonged handling; that is, tail handled animals avoided the handler, tunnel handled animals spent much time in voluntary interaction, while those cupped on the open hand showed intermediate responses (see Experiment 1 Methods). Further, animals handled even briefly using non-aversive methods showed much greater willingness to explore the test arena, which had a major positive impact on test performance. Thus, extensive handling is not necessary to ensure lack of aversion to handling and low anxiety in a novel arena, as long as a suitable non-aversive handling method is used. It appears that this can be achieved through normal brief handling during cage cleaning. Further, the precise schedule of brief handling does not appear to be particularly important. Mice in our first experiment were handled with their assigned method at cage cleaning from three weeks of age and showed clear differences after just five cleaning sessions. Those in the second experiment were obtained as adults and were picked up briefly using their assigned method in ten daily sessions from 14 weeks of age to overcome any prior handling experience (which was unknown but likely to involve traditional tail handling^34^). Despite these differences in prior experience, the effects of handling method on exploratory behaviour and on test performance were very similar between experiments for those in equivalent test groups (i.e. tail or tunnel handled mice with a centralised stimulus and not previously familiarised with the arena). As our study has shown, the key factor influencing poor test performance was reduced exploration and a high degree of caution when placed in the test arena, behaviours that are associated with anxiety^24^,^25^and promoted by tail handling. However, this can be overcome by relatively brief experience of being picked up by non-aversive methods, though the amount of familiarisation required is likely to depend on the animal’s previous experience. Further research will be needed to establish whether more prolonged handling experience has additional benefits.

In our first experiment, animals that were cupped on the open hand or handled with a tunnel showed very similar levels of exploratory behaviour in the test arena. However, while tunnel handled mice showed a robust dishabituation response, with consistently high investigation when urine from a different male was introduced, individual cup handled mice showed greater variation in responsiveness. This may be because tunnel handling is more effective and consistent than cup handling in reducing anxiety to an extent that allows animals to attend to interesting stimuli in their environment. While a handling tunnel allows the animal to satisfy a behavioural need to seek shelter when confronted with a potential threat^37^,^38^, cupping requires the animal to interact directly with the human hand and remain in the open where it is unprotected from potential danger. Mice will voluntarily approach a handling tunnel, even after the first experience of being picked up in the tunnel, suggesting that even the novelty of this experience is not aversive^9^,^10^. However, mice take much longer to become accustomed to being cupped on the open hand. Naïve mice quickly jump off the hand, and willingness to approach the handler increases gradually over multiple handling sessions^9^. A study by Novak and colleagues^39^ found that even following daily cupping for several months, mice failed to show any improvement in performance in the radial maze relative to tail handling. From a practical perspective, delivering mice to a test environment inside a handling tunnel is much safer, particularly for animals that might jump off the hand when unrestrained (e.g. young animals or those with little experience of handling), while their release from the tunnel can be easily standardised by gently tipping animals out backwards.

Importantly, handling tunnels are robust in reducing anxiety-related behaviour across strains and sexes, in the light or dark phase of the diurnal cycle, and are effective even when animals or their handlers have little prior experience of handling^9^,^10^. Use of a non-aversive handling method can remove the need for familiarisation as animals are no longer anxious when introduced to the test. The much greater reliability of behavioural responses to test stimuli, and the safety and practicality of handling mice with a tunnel, strongly indicate that this should be the method of choice to minimise any handling-induced anxiety during behavioural testing.

## Methods

All procedures involved in this study were non-invasive behavioural tests. Animal use and care was in accordance with EU directive 2010/63/EU and UK Home Office code of practice for the housing and care of animals bred, supplied or used for scientific purposes. The University of Liverpool Animal Welfare Committee approved the work, but no specific licenses were required.

### Subjects and handling: Experiment 1

Subjects were 48 female BALB/c mice (BALB/cOlaHsd) purchased from Harlan UK at 3–4 weeks of age, housed in pairs in 43 × 11.5 × 12 cm cages (M3, North Kent Plastics Rochester, UK) on Corn Cob Absorb 10/14 substrate (IPS Product Supplies Ltd, London, UK). This strain shows relatively high anxiety^40^,^41^,^42^, so was likely to be susceptible to effects of handling stress. All mice were provided with a clear acrylic tunnel (150 mm long × 50 mm wide) and paper wool nesting material (IPS Product Supplies Ltd) as sources of home cage enrichment. Water and food (lab diet 5002 certified rodent diet, Purina Mills) were given *ad-libitum*. Animals were housed under a reversed 12:12 h light schedule (lights on 8 pm–8 am) and tested during the active dark period under red lighting.

On arrival, animals were picked up by the tail to transfer them to cages. Pairs of cagemates were assigned randomly to one of three groups that were handled consistently by one of three methods during routine cage cleaning, every two weeks for five cage cleaning sessions before testing at 14–15 weeks of age (n = 16 mice per handling group, mice assigned randomly to cage cards that ensured a balanced design of treatment groups on the cage rack). Mice handled by the tail (traditional method) were picked up by the base of the tail (between thumb and forefingers) to move them into a clean cage (the body was not supported during these short lifts in line with standard practice^34^). Tunnel handled mice were encouraged into the tunnel present in the home cage and moved between cages inside the tunnel with hands covering the ends to prevent egress. Cupped mice were picked up first between closed hands and then on the open hands once mice no longer jumped away (see Hurst & West^9^ for details). In each case, the handling and transfer of animals between cages took approximately 2 s.

In addition, before habituation-dishabituation tests were carried out, we assessed the willingness of mice to interact voluntarily with the handler after they were picked up by their assigned method in the fifth cage cleaning session (animals aged 13–14 weeks, see Hurst & West^9^ for full details of test including movie files). Briefly, after animals had been picked up and transferred to a clean cage by their accustomed method, the handler held a gloved hand in the front half of the open home cage for 60 s (holding a home cage tunnel for the tunnel handled group or hand only for tail or cup handled mice). The proportion of test time that animals spent interacting with the handler (sniffed the gloved hand or tunnel, made paw contact, climbed on, or entered the handling tunnel) was measured from DVD recordings. This was then averaged across the two mice in each cage as they were tested together and their behaviour was not independent (data provided in Supplementary Dataset 1). Mice handled by these three different methods showed substantial differences in voluntary interaction with the handler (ANOVA, F_2,21_ = 10.37, P = 0.001). Mice handled with the home cage tunnel spent a much greater proportion of the test interacting with the handler (mean ± s.e.m., 39.8 ± 5.2 percent time of 60 s test, n = 8 cages) than those handled by tail (6.4 ± 2.0 percent time, n = 8 cages), while those handled by cupping showed intermediate levels of voluntary interaction (27.6 ± 7.1 percent time, n = 8 cages). These differences were in line with those reported by Hurst & West^9^ who used much more prolonged handling of 60 s per session compared to the 2 s used here.

Four animals were withdrawn from the study after this (one tunnel handled and three cup handled mice): one due to development of stereotypy and one due to ill health before behavioural testing; two due to human error running the test procedure. Note that, as handling required the use of different physical procedures, it was not possible to blind handling treatments during testing. Nonetheless, it is unlikely that the pronounced and consistent effects of these handling methods on behaviour (seen also in previous studies^9^,^10^) could be explained by subtle differences in handler or observer bias.

### Subjects and handling: Experiment 2

Subjects were 32 female BALB/c mice obtained from Harlan UK at 12 weeks of age. They were assigned randomly to two groups that were habituated to brief handling using a tunnel in the home cage or picked up by the tail. Animals were obtained as adults so that they could be tested within a shorter timeframe than experiment 1. As handling experience prior to arrival was unknown, animals were familiarised with their assigned handling method at 14 weeks of age in ten daily sessions (including cage cleaning) when they were picked up for 2 s, equivalent to the duration of handling during cage transfer, to overcome their prior experience. Animals were tested in the week following their last handling session (aged 16 weeks). Otherwise, conditions were the same as experiment 1.

### Habituation-dishabituation test procedure

In both experiments, animals were tested individually in an olfactory habituation-dishabituation task using urine from two different male mice as the habituation and dishabituation stimuli. Urine donors were six unrelated singly housed genetically heterogeneous wild-stock male mice to ensure that urine stimuli were easily discriminable and came from donors of equal social status. Urine donors were housed in M3 cages in a different room, with the same husbandry conditions as subjects. Urine samples from different donors were used in a balanced design across experimental groups and as habituation or dishabituation stimuli with different subjects. Urine was obtained by scruffing individual males over a clean Eppendorf tube and stored at −20 °C until use.

Mice were placed into a clean open rectangular arena (70 × 60 × 55 (H) cm, white laminate board) using their assigned handling method, with the test stimulus presented either in the centre of the arena or close to the periphery (Fig. 1b). Tunnel handled mice were tipped out backwards at floor level; cupped mice were gently tipped from the hand. All mice were naïve to being placed in a test arena at the start of the experiment. To induce reduced stimulus investigation towards the same stimulus over repeated presentations (habituation), subjects were presented with 10 μl urine from the same male donor in three consecutive 5 min trials, with a 5 min inter-trial interval. They were then presented with 10 μl urine from a different male to induce increased investigation of the novel stimulus (dishabituation). Urine stimuli were streaked onto clean microscope slides that were stuck to a transparent plastic tile (14.5 × 14.5 cm) with reusable adhesive (Blue tack, Bostik Limited, UK), using fresh stimuli in each trial. During inter-trial intervals, mice were held in a clean empty holding cage (M3, North Kent Plastics). The arena, tile and glass slide were cleaned between trials using 70% ethanol and dried with clean paper tissue. Any excrements left in the holding cages between trials were removed with a dry clean paper tissue. Tests were carried out within the first three hours of the dark phase of the light dark cycle, to ensure that mice were naturally at their most active during testing.

In experiment 1, half of the cages in each handling group were assigned randomly to each stimulus location (centre or periphery, cage-mate pairs assigned to the same handling method and stimulus location). In experiment 2, half of each handling group were assigned randomly to naïve or familiarisation prior to the habituation-dishabituation task. Those assigned to familiarisation were placed in the test arena, using their assigned handling method, and allowed to explore for 10 minutes before being held in a clean empty holding cage while the arena was cleaned for their first habituation trial. No urine stimulus was present during this familiarisation period. Otherwise, the test procedure was identical to experiment 1. However, in experiment 2, all mice were tested with the stimulus in the centre of the arena as this induced the most robust responses in our first experiment.

### Behavioural measures and data analysis

All trials were recorded onto DVD for subsequent analysis. To assess performance in the test, we recorded time spent sniffing the urine stimulus (nose <1 cm from the stimulus) during each trial. To assess general behaviour during testing, we also measured the number of stretched attend postures (animal stretches the front half of its body forward to scan the environment but withdraws again without moving its back feet forwards, a behaviour associated with risk assessment and anxiety^43^,^44^); the number of visits to the stimulus tile; and movement around the arena. Movement was measured by marking out the different areas of the arena (centre, periphery, corners) on an acetate sheet placed over the DVD playback and the number of lines crossed between these areas scored for each trial. The behaviour of each test subject was averaged over the four trial periods for each of these four measures. As the measures were strongly correlated, a principal components analysis was used to extract the main component(s) of exploratory behaviour for further analysis. Data for experiments 1 and 2 are provided in Supplementary Datasets 2 and 3 respectively.

To analyse habituation performance, we tested whether the duration of stimulus investigation was reduced in trial 3 compared to trial 1 (novel urine). For dishabituation performance, we tested whether the duration of stimulus investigation was greater in trial 4 (novel urine) than in trial 3. In both experiments, median response levels for mice in some test groups were zero. Thus, non-parametric tests were used to assess the significance of test performance achieved by each test group. As test performance is clearly directional in both cases (e.g. dishabituation performance is only achieved when trial 4 is greater than trial 3), specific (one-tailed) non-parametric Wilcoxon matched pair signed ranks tests assessed whether animals achieved statistically significant performance measures within each treatment group.

Linear mixed effects models were used to assess the effects of handling method and stimulus location (experiment 1), or handling method and arena familiarisation (experiment 2), on habituation and dishabituation measures of test performance, and on general behaviour across trials (PC1). Home cage was included in each model as a random effect (assuming random intercepts). For each model, we checked that residuals approximated normality (Shapiro Wilk’s tests, P > 0.1, linear Q-Q normality plots) and visual inspection of residuals plots did not reveal any obvious deviations from homoscedasticity or strong outliers. Where necessary, sniffing duration was log transformed to meet these assumptions (required for dishabituation responses in experiment 2). Modelling was carried out using R (version 3.2.5) and the *lme4* test package^45^. Likelihood ratio tests compared the full model against a reduced model without the effect of interest using the anova function. To assess whether treatment effects on general exploratory behaviour (PC1) could explain individual differences in test performance measures, we also tested models with PC1 included as an additional factor.

## Additional Information

**How to cite this article:** Gouveia, K. and Hurst, J. L. Optimising reliability of mouse performance in behavioural testing: the major role of non-aversive handling. *Sci. Rep.* **7**, 44999; doi: 10.1038/srep44999 (2017).

**Publisher's note:** Springer Nature remains neutral with regard to jurisdictional claims in published maps and institutional affiliations.

## Supplementary Material

Supplementary Dataset 1

Supplementary Dataset 2

Supplementary Dataset 3

## Figures and Tables

**Table 1:** Mixed effects modelling of response measures in experiment 1.

Response measures	Habituation		Dishabituation		Exploration (PC1)	
	χ^2^	*P*	χ^2^	*P*	χ^2^	*P*
(**a**) Model: handling method (tail, cup or tunnel) and stimulus location (centre or periphery)						
Handling method	0.18	0.67	8.88	0.003	22.03	<0.0001
Location	1.30	0.25	4.76	0.03	2.24	0.13
(**b**) Model: handling method, stimulus location and PC1						
Handling method	2.39	0.12	0.54	0.46		
Location	0.30	0.59	3.15	0.08		
PC1	7.15	0.008	7.78	0.005		

Likelihood ratio tests compare the full model against a reduced model without the effect of interest (1 d.f. in each case). Home cage was included in each model as a random effect.

**Table 2:** Mixed effects modelling of response measures in experiment 2.

Response measures	Habituation		Dishabituation*		Exploration (PC1)	
	χ^2^	*P*	χ^2^	*P*	χ^2^	*P*
(**a**) Model: handling method (tail or tunnel) and familiarisation (naïve or familiar)						
Handling method	4.16	0.04	8.31	0.004	16.79	<0.0001
Familiarisation	8.11	0.004	0.56	0.45	4.38	0.04
(**b**) Model: handling method, familiarisation and PC1						
Handling method	1.60	0.21	0.43	0.51		
Familiarisation	6.79	0.009	0.05	0.82		
PC1	16.30	<0.0001	5.54	0.02		

Likelihood ratio tests compare the full model against a reduced model without the effect of interest (1 d.f. in each case). Home cage was included in each model as a random effect. *Dishabituation was analysed as the difference in log transformed sniffing responses to meet assumptions of the analysis.
